# Flower Development and Perianth Identity Candidate Genes in the Basal Angiosperm *Aristolochia fimbriata* (Piperales: Aristolochiaceae)

**DOI:** 10.3389/fpls.2015.01095

**Published:** 2015-12-11

**Authors:** Natalia Pabón-Mora, Harold Suárez-Baron, Barbara A. Ambrose, Favio González

**Affiliations:** ^1^Instituto de Biología, Universidad de AntioquiaMedellín, Colombia; ^2^The New York Botanical Garden, BronxNY, USA; ^3^Instituto de Ciencias Naturales, Facultad de Ciencias, Universidad Nacional de ColombiaBogotá, Colombia

**Keywords:** *AGAMOUS-like6*, *APETALA3*, *Aristolochia fimbriata*, *FRUITFULL*, magnoliids, MADS-box genes, perianth, *PISTILLATA*

## Abstract

*Aristolochia fimbriata* (Aristolochiaceae: Piperales) exhibits highly synorganized flowers with a single convoluted structure forming a petaloid perianth that surrounds the gynostemium, putatively formed by the congenital fusion between stamens and the upper portion of the carpels. Here we present the flower development and morphology of *A. fimbriata*, together with the expression of the key regulatory genes that participate in flower development, particularly those likely controlling perianth identity. *A. fimbriata* is a member of the magnoliids, and thus gene expression detected for all ABCE MADS-box genes in this taxon, can also help to elucidate patterns of gene expression prior the independent duplications of these genes in eudicots and monocots. Using both floral development and anatomy in combination with the isolation of MADS-box gene homologs, gene phylogenetic analyses and expression studies (both by reverse transcription PCR and *in situ* hybridization), we present hypotheses on floral organ identity genes involved in the formation of this bizarre flower. We found that most MADS-box genes were expressed in vegetative and reproductive tissues with the exception of *AfimSEP2, AfimAGL6*, and *AfimSTK* transcripts that are only found in flowers and capsules but are not detected in leaves. Two genes show ubiquitous expression; *AfimFUL* that is found in all floral organs at all developmental stages as well as in leaves and capsules, and *AfimAG* that has low expression in leaves and is found in all floral organs at all stages with a considerable reduction of expression in the limb of anthetic flowers. Our results indicate that expression of *AfimFUL* is indicative of pleiotropic roles and not of a perianth identity specific function. On the other hand, expression of B-class genes, *AfimAP3* and *AfimPI*, suggests their conserved role in stamen identity and corroborates that the perianth is sepal and not petal-derived. Our data also postulates an *AGL6* ortholog as a candidate gene for sepal identity in the Aristolochiaceae and provides testable hypothesis for a modified ABCE model in synorganized magnoliid flowers.

## Introduction

**FIGURE 1:**
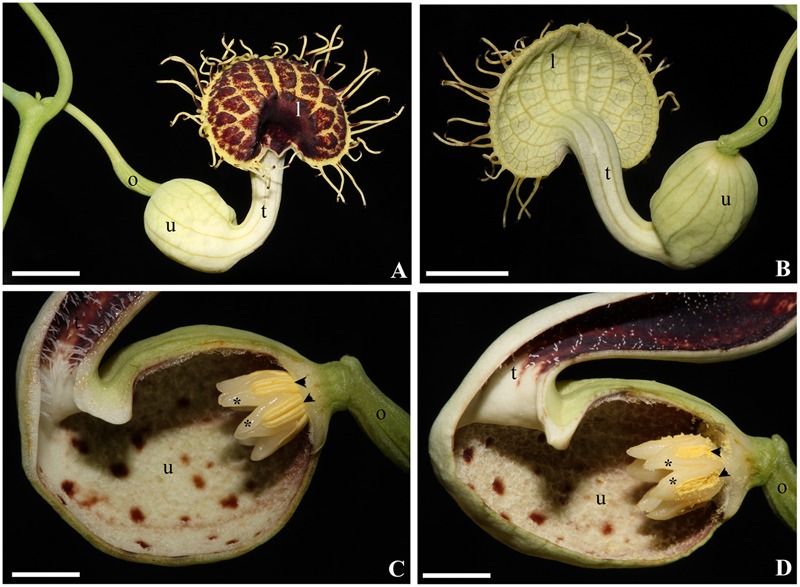
***Aristolochia fimbriata*.** Anthetic flower in face **(A)** and back **(B)** view. Close-up of the gynostemium in female **(C)** and male **(D)** phases. Black arrowheads indicate anthers; asterisks (^∗^) indicate the upper portion of a single carpel alternating with each anther; l, limb; o, ovary; t, tube; u, utricle. Scale bar: 1 cm.

With approximately 550 species, *Aristolochia* is the largest genus in the Aristolochiaceae, one of the families of the monophyletic order Piperales (APG, 2009). Flowers in the genus differ from those of the other members of the family (*Asarum* L., *Thottea* Rottb., *Lactoris* Phil., *Hydnora* Thunb., and *Saruma* Oliver) in that its perianth is monosymmetric, and formed by a trimerous whorl of petaloid sepals fused to form a variously convoluted and tubular structure, differentiated in three regions: a basal, inflated portion called the utricle; a narrow, tubular portion called the tube; and an expanded, laminar portion called the limb (**Figure 1**). All species of *Aristolochia* also possess a gynostemium, that is, a crown-like structure found inside the utricle, above the five or six carpellate, syncarpic, inferior ovary. Ontogenetic studies have shown that the gynostemium is formed by the congenital fusion of stamens and stigmas (González and Stevenson, 2000a,b). This suite of floral characters is unique among early diverging flowering plants, and strongly differ from the predominant floral construction among magnoliids based on a large number of spirally arranged organs and a predominantly apocarpic and superior ovary (Endress and Doyle, 2007). Thus, the floral construction in *Aristolochia* poses a number of questions regarding the origin, development, and genetic identity of whorls in early diverging synorganized flowers.

The genetic basis of floral organ identity was established three decades ago, based on homeotic mutants of *Arabidopsis thaliana* and *Antirrhinum majus* (Schwarz-Sommer et al., 1990; Coen and Meyerowitz, 1991). This resulted in a model that explained how a combinatorial activity of three classes of MIKC MADS-box transcription factors and an *ERF/APETALA2* gene (collectively named A, B, and C class genes) confer a specific identity to each floral whorl. According to the ABC model, A-class genes (*APETALA1* and *APETALA2*) establish the identity of floral meristem and sepals, A and B-class genes (*APETALA3* and *PISTILLATA*) together control petal identity, B and C-class genes (*AGAMOUS*) regulate stamen identity, and finally, C-class genes alone regulate carpel identity, as well as the termination of the floral meristem (Bowman et al., 1989, 1991; Yanofsky et al., 1990). Although the model points to a mutually exclusive role of A and C class genes to control sterile and fertile portions of the flower, there is little evidence that the direct or indirect repression of *AG* via *AP1* or *AP2* orthologs, shown to occur in *Arabidopsis thaliana*, occurs in the same fashion in other flowering plants (Litt, 2007; Dihn et al., 2012). This model was modified when additional transcription factors were found to be required for the initiation of all floral organs, which has resulted in the inclusion of the E-class genes (the *Arabidopsis SEPALLATA1, SEP2, SEP3*, and *SEP4*) in the model (Pelaz et al., 2000). In addition, D-class ovule-specific transcription factors were also identified in *Petunia* and since some authors have proposed an expanded ABCDE model (Pinyopich et al., 2003). However, functional data in other angiosperms, has pointed to little generality of these findings favoring the use of the ABCE model, which we will refer hereafter. MADS-box gene duplications have occurred coinciding with the diversification of the Brassicaceae, the eudicots and the monocots, often linked with whole genome duplication (WGD) events (Jiao et al., 2011). Studies addressed to investigate the genetic control of the diverse floral ground plans in non-model angiosperms have revealed that gene duplications complicate the extrapolation of model to non-model plants, and that functional evolution of different gene lineages involved functions that were not considered in the original model (Litt and Kramer, 2010). Comparative functional studies have demonstrated that, for the most part, stamen and carpel identity are controlled by B and C class genes, in early diverging angiosperms, monocots, magnoliids, and eudicots (reviewed in Litt and Kramer, 2010). In the same way, extra whorls formed outside or inside the stamens, such as the controversial corona in Passifloraceae and *Narcissus* (Hemingway et al., 2011; Waters et al., 2013) are also controlled by the same B + C class gene combination, suggesting that these extra whorls share the same genetic regulation as the stamens themselves.

The genetic basis of perianth identity is, by comparison, more complicated. Petal identity relies on the AP3–PI heterologous interaction with AP1 and SEP proteins in *Arabidopsis thaliana* (Honma and Goto, 2001). Gene duplications in the *AP3* gene lineage have often resulted in the specialization of gene copies in unique structures. For instance, *AP3* gene duplications in core eudicots, resulting in the *AP3* and *TM6* gene clades, have led to sub-functionalization with *AP3* paralogs contributing to the perianth, and *TM6* paralogs functioning in stamen identity (Jack et al., 1994; Liu et al., 2004; Vandenbussche et al., 2004; deMartino et al., 2006; Rijpkema et al., 2006). Independent gene duplication in Ranunculales, have also led to sub-functionalization of *AP3*-*III* homologs exclusively providing petal identity, and their paralogs *AP3-I* and *AP3*-*II* controlling stamen identity (Drea et al., 2007; Kramer et al., 2007; Sharma et al., 2011; Sharma and Kramer, 2012; Zhang et al., 2013). Gene expression patterns and functional analyses of *AP3* homologs have also been used in an attempt to assess homology of atypical floral structures occurring in the second whorl, which are likely modified petals. These include for example the reduced lodicules in grasses (Ambrose et al., 2000; Whipple et al., 2007) and the labellum in orchids (Mondragón-Palomino and Theissen, 2011). On the other hand, petaloidy outside of petals can occur with or without B-class expression. Petaloid sepals in non-grass monocots, like tulips, seem to occur as a result of the ectopic expression of B-class genes in the first floral whorl (Kanno et al., 2007), whereas sepaloid tepals in *Lacandonia* lack B-class gene expression (Alvarez Buylla et al., 2010). Conversely, the identity of petaloid sepals in *Aristolochia manshuriensis* (magnoliids) does not seem to correlate with *AP3* expression, as *ArmAP3* copies are only turned on late in development, exclusively in the adaxial region of the perianth (Jaramillo and Kramer, 2004). Thus, petaloidy is a complex, likely homoplasious feature, both morphologically and genetically and seems to be the result of different gene combinations recruited in a case-by-case scenario (see also Feng et al., 2012; Almeida et al., 2013).

The A-function is by far the most contentious function in the ABCE model. Increasing evidence suggests that *AP1* and *AP2* orthologs rarely control perianth identity outside *Arabidopsis thaliana* (Litt, 2007; Causier et al., 2010). Like the *AP3* and the *AG* gene lineages, the *AP1* gene lineage has undergone duplication events coincident with the diversification of monocots and eudicots, as well as the Brassicaceae (Litt and Irish, 2003). Functional studies suggest that *AP1/FUL* genes are pleiotropic as they are involved in leaf morphogenesis, inflorescence architecture, floral transition, floral meristem identity, and fruit development (Huijser et al., 1992; Gu et al., 1998; Immink et al., 1999; Ferrándiz et al., 2000; Berbel et al., 2001, 2012; Müller et al., 2001; Vrebalov et al., 2002; Murai et al., 2003; Benlloch et al., 2006; Pabón-Mora et al., 2012, 2013). *AP1/FUL* homologs frequently play roles in the identity of the floral meristem and the sepals (Huijser et al., 1992; Berbel et al., 2001; Benlloch et al., 2006) but their contribution to petal identity is unclear (Mandel et al., 1992; Yu et al., 2004; Castillejo et al., 2005; Pabón-Mora et al., 2012). Sepal identity is tightly coupled with the acquisition of floral meristem identity. Potential candidate gene lineages that function in the transition from inflorescence to floral meristem identity, and turn on floral organ identity genes, include the *SEP* and the *AGL6* gene lineages. Functional analyses in model core eudicots and monocots have shown that they function in floral meristem fate and the identity of all floral organs (Pelaz et al., 2000; Ferrario et al., 2003; Ohmori et al., 2009; Rijpkema et al., 2009; Pan et al., 2014). *AGL6* expression has been detected in sepals or paleas (putative first whorl organs) in grasses (Reinheimer and Kellogg, 2009; Viaene et al., 2010) suggesting that in addition to the ABCE genes, the *AGL6* homologs may be controlling fundamental processes in floral organ identity, specifically in floral meristem and sepal identity. This is likely to be the case only in early diverging angiosperms and monocots, as *AGL6* is redundant with *SEP* genes in many core eudicots in the specification of floral organ identity (Rijpkema et al., 2009) or have been co-opted to function in inflorescence architecture and flowering, as is the case in *Arabidopsis* (Koo et al., 2010). In addition, *AGL6* genes are also expressed in ovules (Reinheimer and Kellogg, 2009; Rijpkema et al., 2009) and are the only genes from the *AP1/SEP/AGL6* lineage found in both gymnosperms and angiosperms (Zahn et al., 2005).

The present research aims to assess the genetic basis responsible for the petaloid perianth and the gynostemium identity in *Aristolochia*, having the ABCE model as a reference point (**Figure 1**). In order to do so we have selected *Aristolochia fimbriata* Cham., as this species has recently been proposed as a candidate magnoliid for evolutionary developmental studies for being a self-compatible herb with continuous flowering, and having high rate of seed germination, small genome size, and low (2*n* = 14) chromosome number (Bliss et al., 2013). The species is native to temperate South America but has been widely spread as an ornamental. It has distichous, kidney-shape, variegated, and glabrous leaves, solitary, axillary flowers, and acropetally dehiscent capsules that contain many heart-shaped seeds (González et al., 2015). Here, we present a detailed study of flower development, provide key developmental stages and their associated features, evaluate expression profiles and *in situ* hybridization of the key regulatory genes that participate in floral development, and specifically highlight those that may be responsible for the sepal-derived perianth identity. In particular, we would like to identify perianth expressed genes, as previous studies have found that B-class genes do not play a role in determining the petaloid perianth identity in other species of *Aristolochia* (Jaramillo and Kramer, 2004; Horn et al., 2014). Finally, as *A. fimbriata* is a member of the magnoliids, gene expression detected for all ABCE MADS-box genes can be used to elucidate ancestral patterns of expression for the gene lineages that duplicated during the diversification of eudicots and monocots independently.

## Materials and Methods

### Scanning Electron Microscopy

Floral buds at several stages of development of *A. fimbriata* (Voucher *N. Pabón-Mora* 242, NY) were collected from the living collections at the Nolen greenhouses (NYBG) and at the Universidad de Antioquia (UdeA), fixed in 70% ethanol, and dissected in 90% ethanol. The samples were then dehydrated in a series of 100% ethanol, 50:50% ethanol–acetone, and 100% acetone, critical point-dried using a Samdri 790 CPD (Rockville, MD, USA), coated with gold and palladium using a Hummer 6.2 (Anatech, Springfield, VA, USA) sputter coater, and examined and photographed at 10 kV in a Jeol JSM-5410 LV scanning electron microscope.

### Light Microscopy

For light microscopy, floral buds and mature flowers were also prepared by conventional dehydration with ethanol and toluene using a standard series in a Leica TP-1020 automatic tissue processor and embedded in paraplast X-tra using an AP-280 Microm tissue embedding center; the samples were sectioned at 12 μm with an AO Spencer 820 rotary microtome. Sections were stained in safranin and astra blue, mounted in permount and examined using a Zeiss Compound microscope equipped with a Nixon DXM1200C digital camera with ACT-1 software.

### Isolation and Phylogenetic Analyses of MADS-Box Genes from *A. fimbriata*

Fresh inflorescence and floral tissue from cultivated plants was ground using liquid nitrogen and further total RNA extracted using TRIZOL reagent (Invitrogen). The RNA-seq experiment was conducted using the truseq mRNA library construction kit (Illumina) and sequenced in a HiSeq2000 instrument reading 100 bases paired end reads. A total of 85,608,833 raw read pairs were obtained. Read cleaning was performed with PRINSEQ-LITE with a quality threshold of Q35 and contig assembly was computed using Trinity package following default settings. Contig metrics are as follows: Total assembled bases: 85,608,833; total number of contigs (>101 bp): 118941; average contig length: 719 bp; largest contig: 16972 bp; contig N50: 1823 bp; contig GC%: 42.71%; number of Ns: 0. Orthologous gene search was performed using BLASTN (Altschul et al., 1990) using the *Arabidopsis* sequences as a query to identify a first batch of homologs in the *A. fimbriata* transcriptome. Sequences in the transcriptome were compiled using BioEdit^1^, where they were cleaned to keep exclusively the open reading frame. Ingroup sequences included also MADS-box genes from *Amborella trichopoda* (Amborellaceae), *Saruma henryi* (Aristolochiaceae), *Aquilegia coerulea* (Ranunculaceae), *Mimulus guttatus* (Phrymaceae), and *Arabidopsis thaliana* (Brassicaceae) with the purpose of including at least one species of each major group of eudicots and the ANA grade. Nucleotide sequences were then aligned using the online version of MAFFT^2^ (Katoh et al., 2002), with a gap open penalty of 3.0, an offset value of 1.0, and all other default settings. The alignment was then refined by hand using BioEdit taking into account the conserved MADS protein domain. Outgroup sequences include *SUPRESSOR OF CONSTANS 1* (*SOC1*) copies from basal angiosperms, basal eudicots and core eudicots (Becker and Theissen, 2003; Carlsbecker et al., 2013). Maximum likelihood (ML) phylogenetic analyses using the full nucleotide coding sequences were performed in RaxML-HPC2 BlackBox (Stamatakis et al., 2008) on the CIPRES Science Gateway (Miller et al., 2009). The best performing evolutionary model was obtained by the Akaike information criterion (AIC; Akaike, 1974) using the program jModelTest v.0.1.1 (Posada and Crandall, 1998). Bootstrapping was performed according to the default criteria in RAxML where bootstrapping stopped after 200–600 replicates when the criteria were met. Trees were observed and edited using FigTree v1.4.0 (Rambaut, 2014). Uninformative characters were determined using Winclada Asado 1.62 (Nixon, 2002). Accession numbers for all MADS box genes here identified correspond to KT957081–KT957088.

### Reverse Transcription – PCR (RT-PCR)

Expression of MADS-box homologs was assayed using RT-PCR on RNA extracted from floral buds at four different stages in preanthesis (1.5, 2.5, 3.5, and 4.5 cm) and anthesis. Total RNA was prepared from dissected organs; in this case we separated the perianth from the gynostemium and the ovary and further separated the sepals from the most proximal portions to the most distal ones, into the utricle, the tube and the limb. In addition total RNA was prepared from young leaf and a young fruit (3 cm long); given that fruits in *A. fimbriata* are septicidal capsules with acropetal dehiscence, we chose a green fruit before sclerenchyma accumulation in the endocarp. Total RNA was prepared using TRIzol (Invitrogen, Carlsbad, CA, USA), and DNAseI (Roche, Switzerland) treated to remove genomic DNA contamination. 2 μg were used as template for cDNA synthesis with SuperScript III reverse transcriptase (Invitrogen, Carlsbad, CA, USA). The resulting cDNA was used for PCR-amplification using locus specific primers (Supplementay Table 1), with a thermal cycling regime consisting of one initial step at 94°C for 3 min, 28 cycles at 94°C for 40 s, 55°C for 45 s, and 72°C for 1 min, and a final extension step at 72°C for 10 min. All reactions were carried out in a MultiGene^TM^ OptiMax thermocycler (Labnet International, Edison, NJ, USA). PCR was run on a 1% agarose gel with 1X TAE, stained with ethidium bromide and digitally photographed using a Whatman Biometra BioDocAnalyzer (Gottingen, Germany).

### *In Situ* Hybridization

Developing shoot apical meristems in reproductive stages were collected from wild type plants of *A. fimbriata* growing in the Nolen greenhouses at NYBG or at the Universidad de Antioquia (UdeA), and fixed under vacuum in freshly prepared FAA (50% ethanol, 3.7% formaldehyde, and 5% glacial acetic acid). After an incubation of 4 h, samples were dehydrated in an ethanol series. They were then transferred to toluene and infiltrated with Paraplast X-tra tissue embedding medium (Fisher, Waltham, MA, USA) in a Leica TP1020 automatic tissue processor. Samples were then embedded in fresh Paraplast using a Microm AP280 tissue embedding center and stored at 4°C until use. Samples were prepared and sectioned at 10 μm according to standard methods on a Microm HM3555 rotary microtome. DNA templates for RNA probe synthesis were obtained by PCR amplification of 350–500 bp fragments. To ensure specificity, the probe templates included a portion of the 3′ UTR and the C-terminal portion of the proteins that is specific to each MADS-box gene. Fragments were cleaned using the QIAquick PCR purification kit (Qiagen, Valencia, CA, USA). Digoxigenin labeled RNA probes were prepared using T7polymerase (Roche, Switzerland), murine RNAse inhibitor (New England Biolabs, Ipswich, MA, USA), and RNA labeling mix (Roche, Switzerland) according to each manufacturers protocol. RNA *in situ* hybridization was performed according to Ambrose et al. (2000) and Ferrándiz et al. (2000), optimized to hybridize overnight at 55°C. Probe concentration was identical for all the experiments including the sense control hybridizations. RNase-treated control slides were not used, as expression patterns observed for the target genes were not indicative of artificial signal due to “stickiness” in any particular tissue. *In situ* hybridized sections were subsequently dehydrated and permanently mounted in Permount (Fisher, Waltham, MA, USA). All sections were digitally photographed using a Zeiss Axioplan microscope equipped with a Nikon DXM1200C digital camera.

## Results

### Flower Development

**FIGURE 2:**
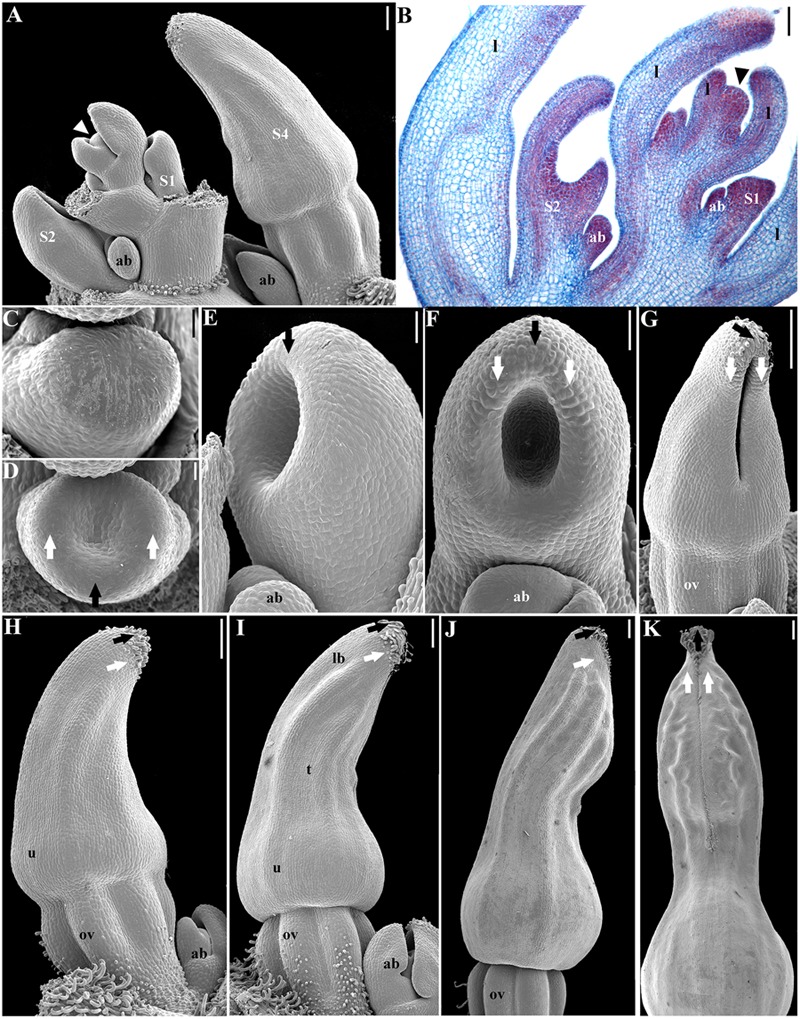
***Aristolochia fimbriata*, perianth development. (A,B)** Shoot apex with floral stages S1 to S4. **(C,D)** Initiation of sepal primordia (stage S1). **(E,F)** Elongation of sepal primordia (S2). **(G)** Perianth closure and ovary (ov) differentiation (S3). **(H)** Initiation of hooked trichomes (S4). **(I)** Differentiation of utricle (u), tube (t), and limb (lb) during S5. **(J,K)** Perianth closure (S6). Black arrows indicate the median sepal; white arrows indicate lateral sepals; arrowheads indicate the shoot apical meristem; ab, accessory bud. Scale bars: **(A,B)**, **(G–K)**: 100 μm; **(C,D)**: 20 μm; **(E,F)**: 40 μm.

**FIGURE 3:**
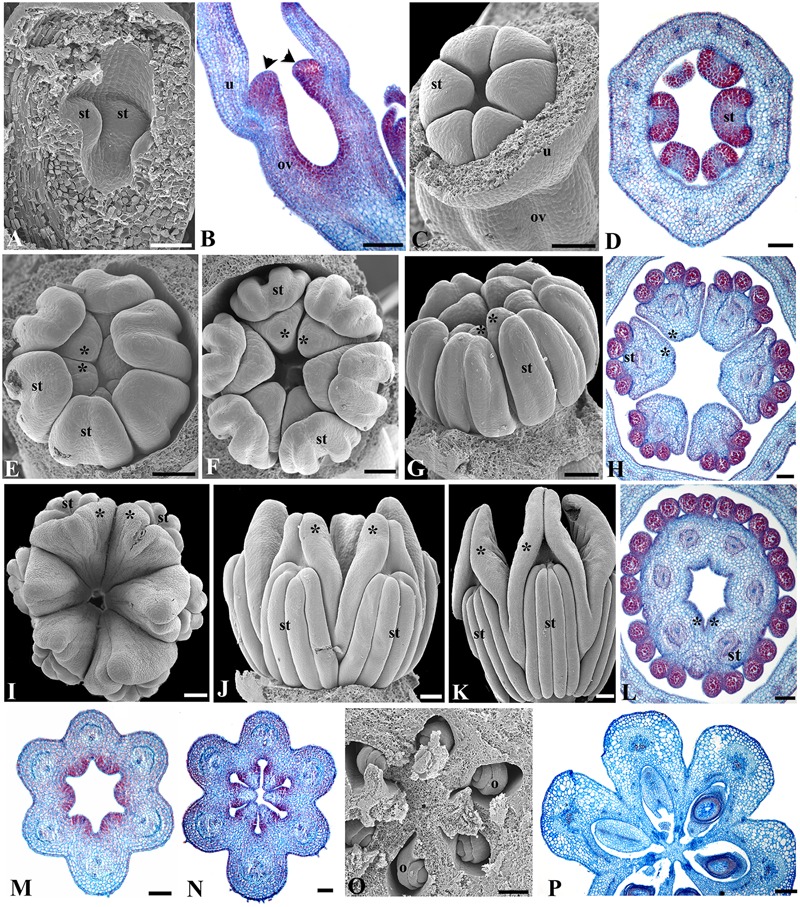
***Aristolochia fimbriata*, gynostemium and ovary development. (A,B)** Stamen initiation above the forming inferior ovary at S3. **(C,D**) Anther growth and formation of the sporogenous tissue at S4. **(E–G)** Two successive stages of gynostemium development at S5 **(E)** and S6 **(F,G)**. **(H)** Transverse section of the gynostemium at S6. **(I–K)** Top **(I)** and lateral **(J,K)** views of the “female” stage of gynostemium with stigmatic lobes growing above the staminal tissue at S7. **(L)** Transverse section of the gynostemium at S7 with carpellary tissue growing between the anthers. **(M–O)** Successive stages of ovary development at S6 **(M,N)** and S7 **(O)**. **(P)** Ovary during transition to fruit **(P)**. o, ovule; ov, ovary; st, stamen; u, utricle; black arrowheads indicate anthers; asterisks (^∗^) indicate stigmas. Scale Bars **(A,B,L)**: 60 μm; **(C,E–G,J,O,P)**: 100 μm; **(D,H,M)**: 50 μm; **(I)**, 200 μm; **(K)**, 300 μm; **(N)**, 80 μm.

The flowering shoots of *A. fimbriata* are indeterminate. A solitary flower is formed axillary to each leaf. An accessory bud is often produced per node, in an adaxial position with respect to the floral bud (**Figures 2A,B**). Floral primordia are monosymmetric, transversally oblong (**Figure 2C**). Flower development can be readily divided into nine different stages prior to anthesis (**Table 1**). Stage 1 (S1) in flower development is here defined by the initiation of two lateral and one median sepal primordia (**Figure 2D**). Next, at stage 2 (S2) the individual sepal primordia become fused in a dome-shaped perianth, which begins to undergo asymmetric intercalary growth, as the abaxial region corresponding to the median sepal grows noticeably faster (**Figures 2E,F**). The rapid elongation of the abaxial region of the growing perianth is likely due to faster cell division rather than cell expansion, as cell size is the same in all flanks of the perianth (**Figures 3A,B**). Stage 3 (S3) is defined by the formation of the furrow between the flanks of the two lateral sepals, and the initiation of the perianth curvature, the anthers and the ovary (**Figures 2G and 3A,B**). Perianth curvature occurs as a result of a stronger elongation of the abaxial flank. At S3 initiation of trichomes in the apex of the growing perianth occurs (**Figure 2G**), and the six anther primordia become evident at the bottom of the perianth above the forming inferior ovary (**Figures 2G and 3A,B**). Stage 4 (S4) can be distinguished by the emergence of hooked trichomes in the outer epidermis of the ovary (**Figure 2H**), the growth of the six anthers and the thecae differentiation (**Figures 3C,D**). By Stage 5 (S5), the future abscission zone between the perianth and the inferior ovary is formed, and a proper utricle, tube and limb can be distinguished (**Figure 2I**); simultaneously, the six stigmatic lobes gradually elongate fused to the inner side of the anthers, overtopping them, thus forming the gynostemium (**Figures 3E–H**). At this stage, the anthers become tetrasporangiate; the filaments never differentiate (**Figures 3E–H**). At Stage 6 (S6), perianth growth and elongation continues and the furrow left by the two lateral sepals closes by a tight interlocking of the marginal epidermis (**Figures 2J,K**); at this stage the total length of the perianth reaches ca. 0.8 mm and the three parts of it (the basal *utricle*, the narrow *tube*, and the *limb*) are already apparent (**Figures 2J,K and 6A**).

**Table 1:** Developmental landmarks for each stage identified during flower development.

STAGE	Developmental landmarks
S1	• Initiation of the sepal primordia
S2	• Sepal fusion • Sepal growth lead by the median sepal
S3	• Furrow formation between the lateral sepals • Initiation of perianth curvature • Inception of the anther primordia • Ovary differentiation
S4	• Differentiation of hooked trichomes on the ovary • Thecae differentiation
S5	• Abscission zone formation between the perianth and the inferior ovary • Perianth differentiation into utricle, tube and limb • Differentiation of the upper portion of the carpels • Fusion between the differentiating carpelar portion and the inner surface of the anthers • Differentiation of the tetrasporangiate anthers
S6	• Closure of the furrow between the two lateral sepals with interlocking epidermis
S7	• Growth of the six stigmatic lobes above the anthers • Ovule initiation (nucellus apparent)
S8	• Resupination of the flower by torsion of the peduncle • Pigmentation initiation in the tube and limb
S9	• Differentiation of the two integuments in the ovules
Anthesis	• Expansion of the limb

**FIGURE 4:**
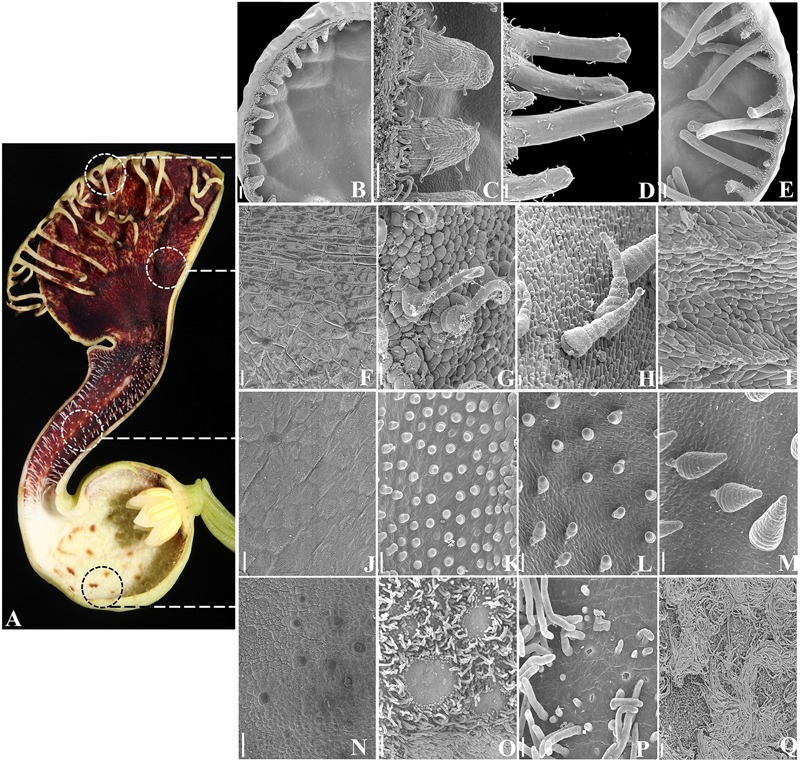
***Aristolochia fimbriata*, epidermal differentiation of the perianth. (A)** Preanthethic flower, longitudinal section. **(B–E)** Fimbriae development and ornamentation. **(F–I)** Ornamentation of the abaxial **(F)** and adaxial **(G–I)** epidermis of the limb. **(J–M)** Ornamentation of the abaxial **(J)** and adaxial **(K–M)** epidermis of the tube. **(N–Q)** Ornamentation of the abaxial **(N)** and adaxial **(O–Q)** epidermis of the utricle. Scale bars: **(B,E)**: 200 μm; **(C,D,O)**: 100 μm; **(F–N,P)**: 20 μm; **(P)**: 50 μm.

Prior to anthesis, the young flower undergoes drastic changes that include the growth of the six stigmatic lobes above the level of the anthers, a feature that marks the beginning of Stage 7 (S7; **Figures 3I–K and 6B**). The six anthers become totally fused with the stigmatic lobes. The commissural origin of each stigmatic lobe is supported by the presence of two bulges toward their apices, whereas the apex of each carpel remains subterminal and alternate with the stamens (**Figures 3I–L**). In the ovary, the protrusion of the six placentae is simultaneous with the differentiation of two rows of ovule primordia, each formed at the margin of each carpel (**Figures 3M,N**). Stage 8 (S8) is here defined by the repositioning of the flower through resupination, that is, the future floral entrance turns away from the shoot axis as the result of the gradual torsion of the peduncle (**Figures 1A and 6C**). Additionally, at this stage the inner epidermis of the perianth acquires a dull-purple pigmentation in the tube and the limb, while the outer epidermis changes from green to pale yellow (**Figures 4A and 6C**). At Stage 9 (S9; **Figure 6D**) the limb remains closed, the stigmatic lobes are already expanded and wet, and the anatropous, bitegmic, and crassinucellar ovules are fully developed (**Figures 3O,P**).

Anthesis occurs acropetally with one flower opening at a time (**Figures 1A and 6E**). During anthesis, the limb unfolds and expands, displaying the fimbriae and allowing insects to reach the utricle through the tube. At day 1 of anthesis, the gynostemium remains at its “female stage” as wet stigmas are fully expanded and receptive, whereas the anthers are still indehiscent; at day 2–3, the gynostemium enters its “male” stage, recognized by the closure of stigmatic lobes and the dehiscence of the anthers; at this stage the trapped insects are exposed to the pollen (**Figures 1C,D**). By late anthesis, perianth withers and allows the insects to escape (González and Pabón-Mora, 2015).

### Epidermis and Trichomes of the Perianth

The outer epidermis of the utricle, the tube and the limb in *A. fimbriata* is homogenously formed by flat epidermal cells with interspersed stomata, and lacks trichomes (**Figures 1B,C and 4F,J,N**). This contrasts with the much more elaborated inner epidermis, as at least four different types of trichomes develop (**Figures 4A,G–I,K–M,O–Q**). Conical trichomes in the tube develop first at the beginning of S5 (**Figures 4K–M**); these trichomes are secretory during late preanthesis (S8 and S9), but during anthesis they function as the guard trichomes that keep pollinators temporarily trapped. Next, epidermal elaboration in the utricle occurs at S6, as a carpet of long, multicellular, filamentous, nectarial trichomes develop and surround small patches of osmophores and nectarioles (**Figures 4O–Q**). The marginal fimbriae begin to form at S6 (**Figures 4B,C**), and by late preanthesis (S8 and S9; **Figures 4D,E**) they reach their final size but remain folded. They are vascularized, and possess secretory tips with osmophores, and hooked trichomes scattered along their proximal half (**Figures 4B–E**). Most of the inner epidermis of the limb is formed by osmophores, accompanied by scattered conical, and hooked trichomes (**Figures 4G–I**).

### Isolation and Expression of MADS-Box Genes

**FIGURE 5:**
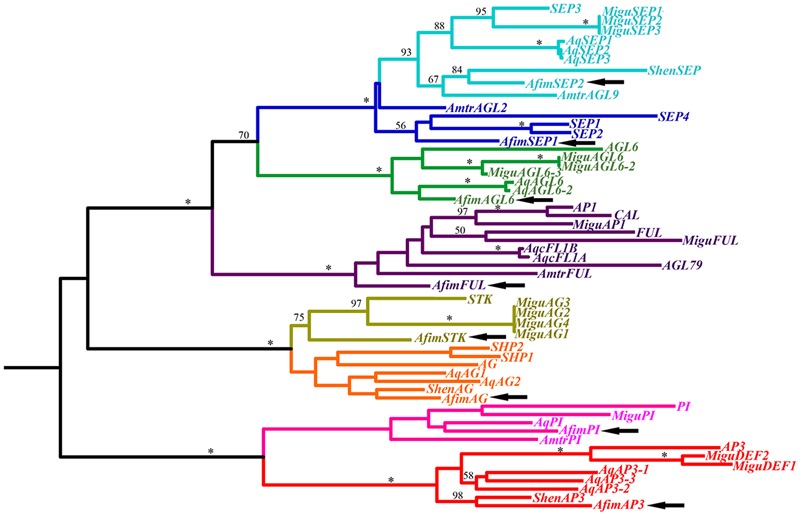
**Maximum likelihood tree of MADS-box genes identified in the transcriptome of *A. fimbriata* (arrows).** Branch colors denote gene clades, as follows: blue, *SEPALLATA*; green, *AGAMOUS*-*Like 6;* purple, *APETALA1/FRUITFULL;* ochre, *SEEDSTICK*; orange, *AGAMOUS*/*SHATTERPROOF;* pink, *PISTILLATA*; red, *APETALA3*. BS values above 50% are placed at nodes. Asterisks indicate bootstrap (BS) of 100. Besides *A*. *fimbriata*, ingroup sequences include those of *Amborella trichopoda* (Amborellaceae), *Saruma henryi* (Aristolochiaceae), *Aquilegia coerulea* (Ranunculaceae), *Mimulus guttatus* (Phrymaceae), and *Arabidopsis thaliana* (Brassicaceae), representing the ANA grade, the magnoliids, the basal eudicots, the asterids and the rosids, respectively.

In order to identify orthologs of the ABCE genes involved in organ identity as well as *SEEDSTICK* and *AGAMOUS-like6*, we searched the generated transcriptome using as a query orthologous genes previously identified from other basal angiosperms (Kim et al., 2005; Yoo et al., 2010). We were able to obtain hits for gene members representing major gene lineages and were able to identify one *AP1/FUL* gene (named *AfimFUL*), two *LOFSEP/SEP3* genes (named *AfimSEP1* and *AfimSEP2*), one *AGL6* gene (named *AfimAGL6*), one *AP3/DEF* gene (named *AfimAP3*), one *PI/GLO* gene (named *AfimPI*), one *AG* gene (named *AfimAG*), and one *STK* gene (named *AfimSTK*; **Figure 5**).

**FIGURE 6:**
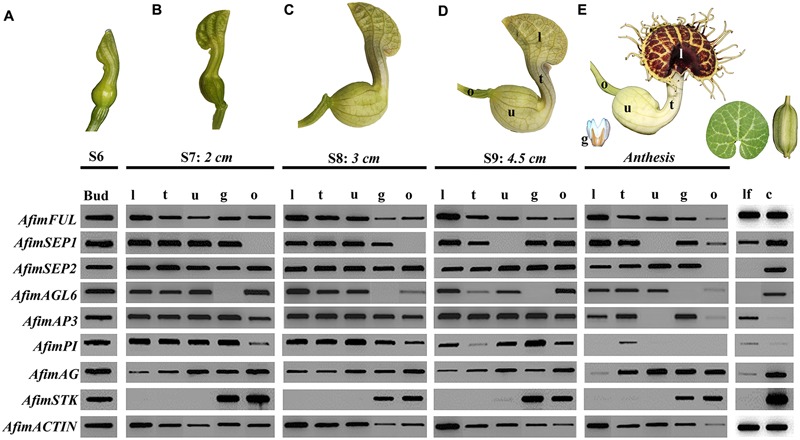
**Expression profiles of *AfimFUL, AfimSEP1, AfimSEP2, AfimAGL6, AfimAP3, AfimPI, AfimAG*, and *AfimSTK* at five different flower developmental stages **(A–E)**, leaves (lf) and capsules (c).** *ACTIN* (*AfimACT*) was used as a positive control. Measurements below each stage correspond to the total length of the perianth. From S7 onward **(B)**, flowers were dissected into gynostemium (g), limb (l), ovary (o), tube (t), and utricle (u).

In order to investigate the expression patterns across developmental stages of all genes involved in the ABCE model of flower development as well as *STK* and *AGL6* we did an expression screening using reverse transcription (RT)-PCR (**Figure 6**). We tested the expression of all copies at different developmental stages from S6 through anthesis. In addition, we tested gene expression in leaves and capsules. Our results show that most genes are expressed in vegetative and reproductive tissues with the exception of *AfimSEP2, AfimAGL6*, and *AfimSTK* transcripts that are only found in flowers and capsules but are not detected in leaves (**Figure 6**). Two genes show ubiquitous expression. *AfimFUL* (the putative “A-class” gene, ortholog of *AP1* and *FUL*) that is found in all floral organs at all developmental stages as well as in leaves and capsules, and *AfimAG* (a “C-class” gene ortholog of *AG* and *SHP1*/*2*) that has low expression in leaves and is found in all floral organs at all stages with a considerable reduction of expression in the limb of anthetic flowers (**Figure 6**). The two E-class gene copies *AfimSEP1* and *AfimSEP2* show different expression patterns; *AfimSEP1*, (ortholog of *SEP1/2/4*) is expressed in all floral organs at early stages S6, S7, and S8 except in the ovary and turned off specifically in the utricle of the perianth at S9 and anthesis, stages at which is present in the ovary; in contrast, *AfimSEP2* (ortholog of *SEP3*) is found in all floral organs throughout development and its expression is only reduced in the ovary of the flowers at anthesis (**Figure 6**). *AfimAGL6* is detected in the perianth at all stages. *AfimAGL6* is never expressed in the gynostemium. In addition, the expression of *AfimAGL6* in the ovary is reduced from S9 to anthesis (**Figure 6**). The B-class gene copies *AfimAP3* and *AfimPI* are both widely expressed in all floral organs at stages S7–S9, however, at anthesis *AfimAP3* becomes restricted to the limb and the tube in the perianth and to the gynostemium, whereas *AfimPI* is only expressed in the tube at very low levels (**Figure 6**). Out of all genes evaluated, both *AfimAP3* and *AfimPI* are the only two genes that are not expressed in the capsules (**Figure 6**). Finally the D-class gene *AfimSTK* is only found in the gynostemium and the ovary during all stages of flower development and its expression is persistent in the capsule (**Figure 6**).

### Detailed Expression Analyses of Perianth Identity Candidate Genes

**FIGURE 7:**
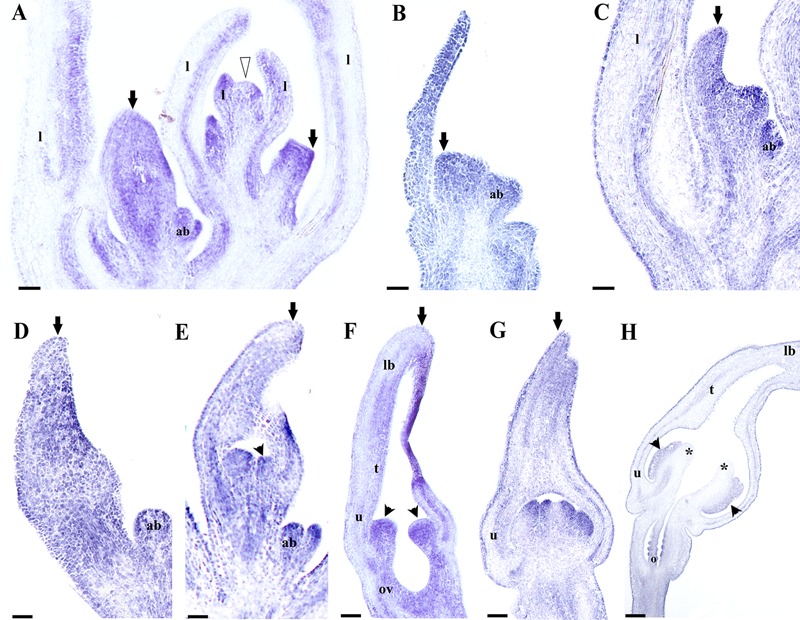
***In situ* hybridization of *AfimFUL.* (A)** Flowering shoot apex; note broad expression in floral stages S1 through S4. **(B)** Floral primordium. **(C,D)** Flower buds during perianth initiation and early elongation at S1–S2. **(E,F)** Stamen initiation at S3 **(E)** and elongation at S4 **(F)**; note broad expression in perianth and gynostemium. **(G,H)** Gynostemium and ovary differentiation at S5 **(G)** and S7 **(H)**; note late expression (in **H**) restricted to perianth, anthers, and ovary. Black arrows indicate medial sepal; black arrowheads indicate anthers; white arrowhead indicates shoot apical meristem (SAM); asterisks indicate stigmas; ab, accessory bud; l, leaf; ov, ovary; o, ovules. Scale bars: **(A–C)**: 100 μm; **(D–H)**: 200 μm.

With the purpose of identifying putative perianth identity candidate genes, in this case sepal-derived, we decided to investigate the expression of *AfimFUL* using *in situ* hybridization experiments to evaluate its contribution to the initiation and development of the perianth parts, as it is a member of the *AP1/FUL* gene lineage to which sepal and petal identity has been attributed in *Arabidopsis* as well as in more early diverging lineages such as *Papaver* and *Eschscholzia* (Papaveraceae, basal eudicots; Bowman et al., 1991; Kempin et al., 1995; Pabón-Mora et al., 2012). Our results are consistent with the RT-PCR and show that expression of *AfimFUL* is very broad and can be detected in the shoot apex, young and old leaves, floral meristems, as well as accessory buds and their respective bracts (**Figures 7A,B**). However, *AfimFUL* expression shifts from a homogeneous expression in young leaves to a localized adaxial expression in older leaves (**Figure 7A**). *AfimFUL* is detected throughout flower development between stages S1–S6, starting with a broad expression during the differentiation of sepal primordia (S1; **Figure 7C**) that is maintained during their asynchronous growth (S2, 3; **Figures 7E–G**), with a higher expression in the adaxial perianth region between the upper or medial sepal and the two lower sepal lobes (**Figures 7D–F**). *AfimFUL* transcripts are also present in the stamen primordia since their initiation and until pollen differentiation (S3–S6; **Figures 7E–H**). Despite this broad expression, *AfimFUL* was not detected in the stigmatic portion of the carpels at the gynostemium (S5, S6; **Figures 7G,H**). At S6 (the oldest stage analyzed using *in situ* hybridization) *AfimFUL* is expressed in the perianth, in particular in the inner and outer epidermis and the vasculature, the stamens, the ovary and the ovule primordia.

**FIGURE 8:**
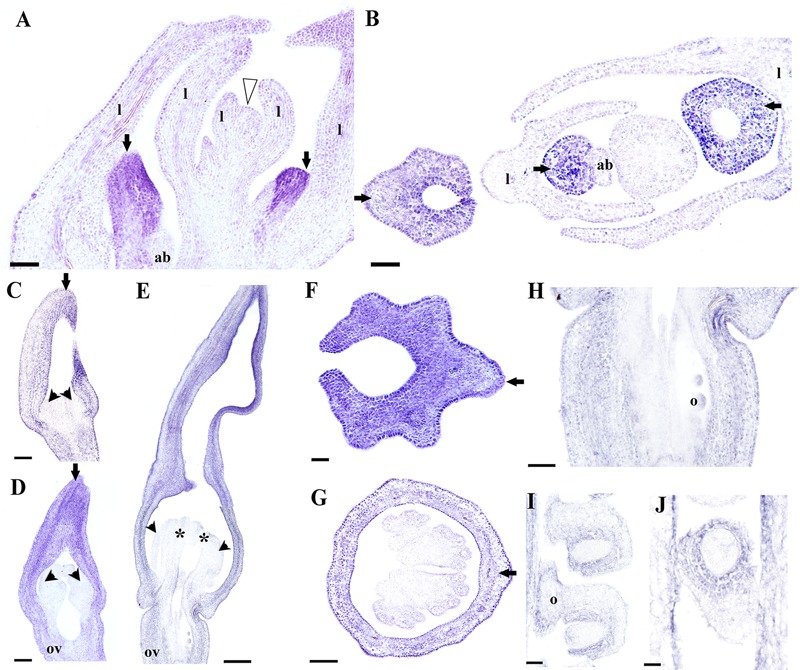
***In situ* hybridization of *AfimAGL6*. (A,B)** Flowering shoot apex in longitudinal **(A)** and cross **(B)** sections; note onset of expression in the perianth. **(C–E)** Successive stages S3–S6 showing stamen initiation at S3 **(C)**, stamen elongation at S4 **(D)**, and ovary development with ovule differentiation at S7 **(E)**; note expression restricted to perianth and ovary. **(F,G)** Cross- sections of a floral bud at the limb **(F)** and the utricle/gynostemium **(G)** levels; note expression restricted to the perianth. **(H)** Longitudinal section of the ovary. **(I,J)** Longitudinal **(I)** and cross **(J)** sections of the ovules; note expression in the ovary wall and the integuments. Black arrows indicate medial sepal; black arrowheads indicate anthers; white arrowhead indicates shoot apical meristem; asterisks indicate stigmas; ab, accessory bud; l, leaf; o, ovules; ov, ovary. Scale bars: **(A,B,E)**: 100 μm; **(C,D,F,G)**: 50 μm; **(H–J)**: 60 μm.

Next, we decided to analyze *AfimAGL6* detailed expression, which according to our RT-PCR is detected in the perianth during flower development but it is never detected in the gynostemium. Contrary to *AfimFUL*, the expression of *AfimAGL6* is localized to the perianth early on during the initiation of the three sepal primordia (S1; **Figures 8A,B**) and throughout flower development (S2–S6; **Figures 8C–G**). *AfimAGL6* is not detected in the shoot apex, the leaves or the axillary dormant buds (**Figures 8A,B**). Its expression in the perianth is continuous from the utricle to the limb (**Figures 8C–G**). *AfimAGL6* is not detected in the stamen primordia early on (S3, S4; **Figures 8C,D**) or in the young gynostemium (S5, S6; **Figure 8E**) nor in the staminal or the stigmatic tissue. *AfimAGL6* is turned on again in the pedicel at the level of the ovary since S3 and then later on at S6 in the ovule primordia (**Figures 8C–E,H**). Late expression of *AfimAGL6* is detected in the outer and the inner integuments of the ovule (**Figures 8I,J**).

**FIGURE 9:**
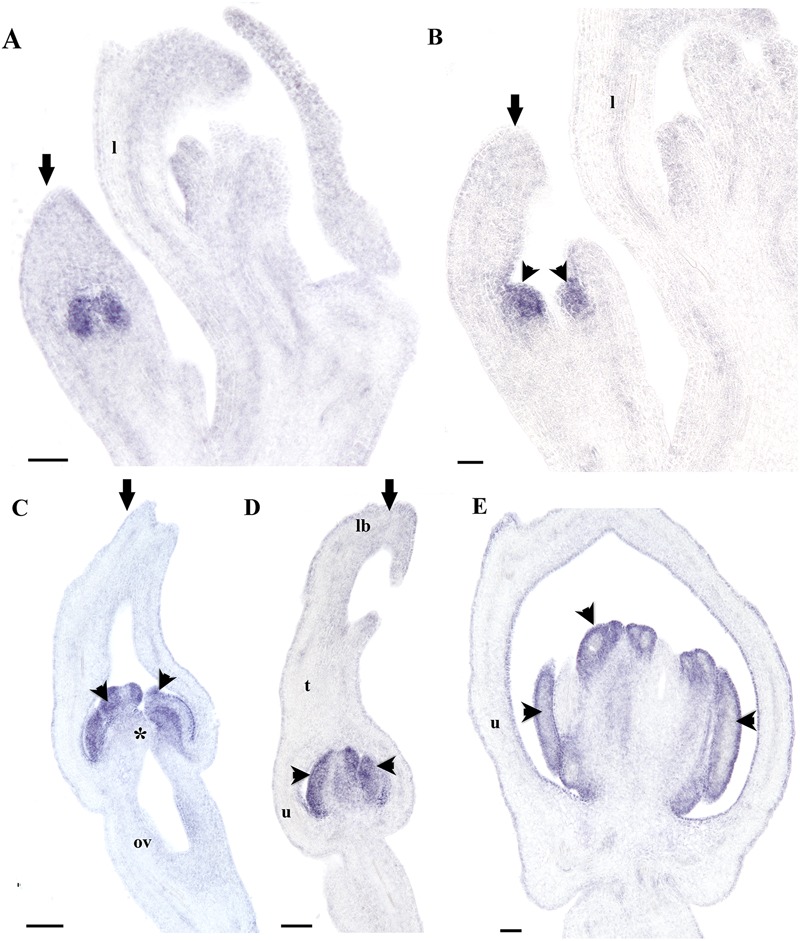
***In situ* hybridization of *AfimAP3*. (A,B)** Flowering shoot apex in longitudinal sections; note expression restricted to stamens. **(C–E)** Successive stages S5-S7 showing initiation of the stigmatic lobes at S5 **(C)**, and elongation at S6–S7 **(D,E)**; note expression restricted to stamens. Black arrows indicate medial sepal; black arrowheads indicate anthers; asterisk (^∗^) indicates a stigma; l, leaf; lb, limb; ov, ovary; t, tube; u, utricle. Scale bars: **(A–D)**: 100 μm; **(E)**: 150 μm.

Finally, we decided to investigate the contribution of the B-class genes to perianth identity in *A. fimbriata*. The expression of *AfimAP3* detected through *in situ* hybridization is also very broad, which is consistent with the RT-PCR results, and the transcripts are present at low levels in the shoot apex, leaves and floral meristems (**Figures 9A,B**). Expression of *AfimAP3* increases at S3 and S4, when it is localized to the stamen primordia (**Figures 9A,B**). Expression of *AfimAP3* continues to be localized in staminal tissue in the gynostemium (S5, S6; **Figures 9C–E**), but it is not detected in the stigmatic portion of the gynostemium or in the ovary (**Figures 9C–E**).

**FIGURE 10:**
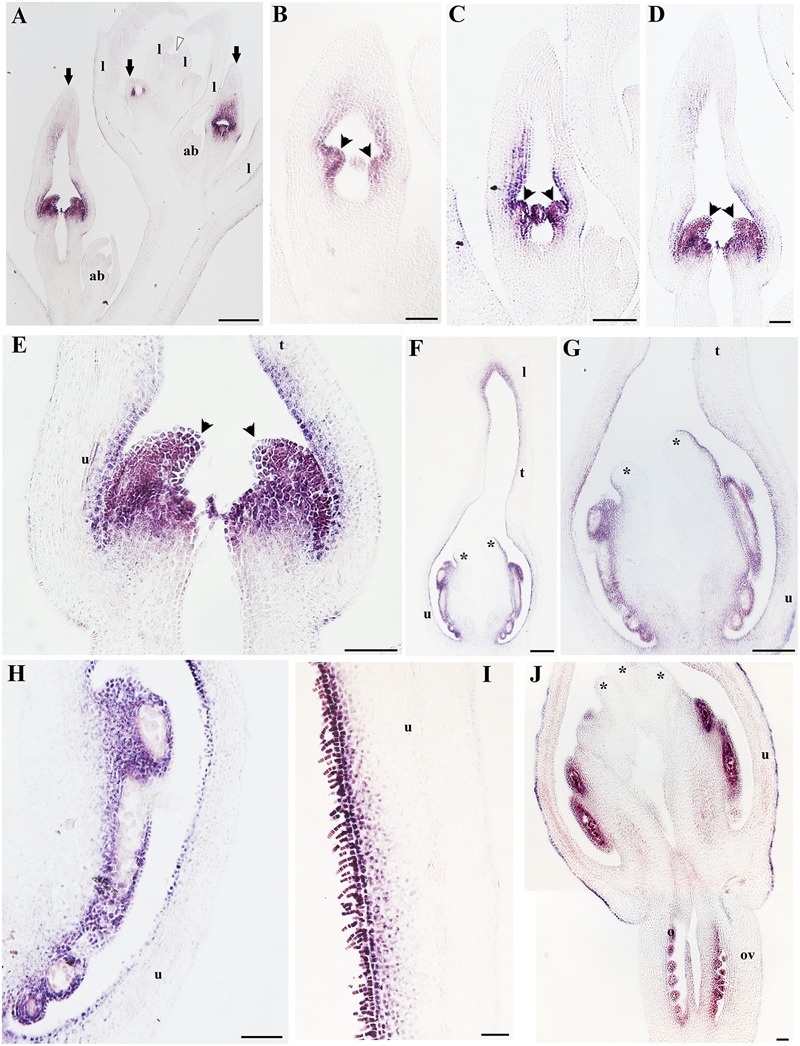
***In situ* hybridization of *AfimPI*. (A)** Flowering shoot apex in longitudinal section; note expression in stamens and surrounding perianth tissue. **(B)** Stamen initiation at S3. **(C)** Stamen growth at S4. **(D,E)** Utricle, tube and limb differentiation together with stigmatic tissue initiation at S5; note expression in the young gynostemium. **(F–J)** Longitudinal sections and details of a floral bud at the utricle/gynostemium level at S7 **(H)** and the distal-most adaxial portion of the utricle **(I)**; note expression in the adaxial epidermis, trichomes and hypodermis. Black arrows indicate medial sepal; black arrowheads indicate anthers; white arrowhead indicates shoot apical meristem (SAM); asterisks indicate stigmas; ab, accessory bud; l, leaf; o, ovules; ov, ovary. Scale bars: **(A–D,F,G)**: 100 μm; **(E)**: 150 μm; **(H,I,J)**: 50 μm.

On the other hand, *AfimPI* is broadly expressed in the perianth, the gynostemium and the ovary according to the RT-PCR (**Figure 5**). However, its expression turns off almost completely in all floral organs as the flower enters anthesis (**Figure 5**). *In situ* hybridization results confirm that *AfimPI* is turned on at S3 in stamen primordia and maintained there until S6–S7 (**Figures 10A–C**). In addition, during S3 *AfimPI* is also expressed in the emerging gynostemium lobes and in the inner cell layers of the perianth starting in the utricle, and expanding toward the tube and limb (**Figures 10C–G**). By S5–S6, expression of *AfimPI* expands to outer staminal portion of the gynostemium and the inner layers of the base of the perianth, and later to the distal portion of the utricle that limits with the tube (**Figures 10H,I**). By S6 *AfimPI* becomes restricted to the fertile portions of the stamens and is turned on in the ovules (**Figure 10J**).

## Discussion

The Aristolochiaceae encompasses an enormous variation of life forms and floral morphologies including flowers with radial (*Saruma, Asarum*, and *Thottea*) as well as bilateral symmetry (*Aristolochia*), with (*Saruma*), or without petals (*Asarum, Thottea*, and *Aristolochia*), and having partial (*Saruma, Asarum, Thottea*) or total congenital fusion between stamens and carpels to form a gynostemium (*Aristolochia*). Following such character combination, *Aristolochia* species are unique in possessing deep, tubular, sepal-derived perianths with strong monosymmetric, highly synorganized flowers (González and Stevenson, 2000a,b; González and Pabón-Mora, 2015). *A. fimbriata* produces 6–7 floral plastochrones from the shoot apex that can include all early stages (from S1–S6) in less than a full centimeter (**Figures 2, 3**). *Aristolochia fimbriata*, like most *Aristolochia* species, undergoes early fusion of the sepals followed by asymmetrical growth of the perianth (S2–S4), almost simultaneously with the fusion between anther primordia and the upper portion of the carpels (S3–S4). In addition, *A. fimbriata* differentiates the utricle, tube and limb with distinct unique epidermal specializations (S5–S6). Thus, *A. fimbriata*, serves as a unique reference point for studying complex floral features like organ fusion, and extreme synorganization and transfer of function from the suppressed petals to a sepal-derived perianth.

### *Aristolochia fimbriata* has a Floral MADS Box Gene Toolkit Similar to Other Early Divergent Angiosperms

According to the APG (2009), the magnoliids are more closely related to the earliest diverging angiosperms Amborellales, Nymphaeales, and Austrobaileyales and altogether these lineages were thought to have evolved prior to the diversification of monocots and eudicots. More recently, the phylogenomic approach using whole transcriptome data (oneKP project), has resulted in an alternative topology, with the magnoliids as sister to the eudicots only, and the monocots evolving independently (Wickett et al., 2014; Zeng et al., 2014). WGD events have been proposed to occur at different times during plant evolution, once, prior to the diversification of angiosperms (ε), two times in the monocots (ρ, σ), once before the diversification of the eudicots (γ), and twice in the Brassicaceae (α, β; Jiao et al., 2011). In addition to these large-scale duplications, *K*s distributions of paralogs have been used to propose several additional basal-angiosperm – specific genome duplications, one shared between Laurales and Magnoliales and another within Piperales only (Cui et al., 2006). These studies raise the question regarding the genetic complement present in basal angiosperms, and in particular in Piperales with respect to the earliest diverging angiosperms on one side and the specious monocot and eudicot clades, on the other. Our results show that *A. fimbriata* has a similar floral genetic toolkit to that found in the earliest diverging ANA members, *Amborella trichopoda* (Amborellaceae) and *Nuphar pumila* (Nymphaeaceae; Amborella Genome Project, 2013; Li et al., 2015). The *A. fimbriata* flower and fruit mixed transcriptome allowed us to find expression of a single copy of each A, C, D-class, and AGL6 MADS-box clade, and two paralogs for the B and the E-class genes, which are known to have duplicated prior to the diversification of angiosperms likely in the ε WGD event (**Figure 5**; Kramer et al., 1998; Becker and Theissen, 2003; Zahn et al., 2005). These results suggest that no additional duplications or losses have occurred in *A. fimbriata* when compared to *Amborella trichopoda*, the earliest diverging angiosperm. Nevertheless copy number can only be confirmed with genome sequencing and broader phylogenetic samplings will have to be done to assess whether duplications have occurred in other magnoliids independently. This approach is critical, given their phylogenetic affinities with the eudicots under the most updated plant classification system, and considering that other analyses have shown taxa specific duplications for *Persea americana* (Laurales) and *Liriodendron tulipifera* (Magnoliales; Cui et al., 2006; Wickett et al., 2014; Zeng et al., 2014).

### Floral Meristem and Perianth Identity are Likely Determined by *FUL-like* and *AGL6* Genes in *A. fimbriata*

Our evaluation of the expression of A-class and *AGL6* genes during *A. fimbriata* flower development indicates that *AfimFUL* and *AfimAGL6* have homogeneous overlapping expression in the sepal primordia (S1) and during perianth fusion and elongation (S2–S4). Moreover, at later stages of flower development, *AfimFUL* expression expands to all other floral organs, including ovules, whereas *AfimAGL6* is expressed only in carpels and ovules (**Figures 7** and **8**). *AfimFUL* is also expressed in the shoot apical meristem and leaves during development (**Figure 7**). Our data are consistent with qRT-PCR expression data shown for *FUL* and *AGL6* homologs in other magnoliids and basal angiosperms (Kim et al., 2005; Yoo et al., 2010). In addition, our *in situ* hybridization results provide a better assessment of spatio-temporal expression patterns and more accurate predictions of putative functions associated with the activation of these transcription factors.

*AfimFUL* is part of the *AP1*/*FUL* gene lineage, while *AfimAGL6* is part of the *AGL6* gene lineage, the former is angiosperm specific, whereas the latter, is present in all seed plants (Litt and Irish, 2003; Viaene et al., 2010; Kim et al., 2013). The original ABCE model in *Arabidopsis* established that *AP1* (in the *AP1/FUL* gene lineage) was responsible for floral meristem and perianth identity (Coen and Meyerowitz, 1991). Gene evolution analyses, coupled with expression and functional studies of *AP1/FUL* homologs across angiosperms has revealed a complex scenario of two rounds of gene duplication, resulting in the *euFULI, euFULII*, and *euAP1* clades in core eudicots, accompanied by subfunctionalization (Litt and Irish, 2003; Pabón-Mora et al., 2012). Specifically, *euAP1* and *euFUL* core-eudicot paralogs, have non-overlapping expression patterns consistent with their unique roles in plant development. While *euAP1* genes including the canonical *AP1*, are turned on in floral meristems and perianth, and function determining sepal (and sometimes petal) identity, *euFUL* genes are expressed during the transition to inflorescence meristem and later on in the carpel and fruit and control the transition from inflorescence to flower as well as proper fruit wall development (Huijser et al., 1992; Kempin et al., 1995; Gu et al., 1998; Ferrándiz et al., 2000). The pre-duplication *FUL-like* genes have also undergone local duplications (Litt and Irish, 2003). Functionally, *FUL-like* genes have been characterized in grasses (monocots), where they play roles in the transition to reproductive meristems (Murai et al., 2003), and the Ranunculales (basal eudicots), where they function in flowering time, patterning inflorescence architecture, leaf morphogenesis, floral meristem and sepal identity, late petal epidermal differentiation and fruit development (Pabón-Mora et al., 2012, 2013; Sun et al., 2014). The broad expression patterns of *AfimFUL* suggest that the pleiotropic roles of *FUL-like* genes occur in *Aristolochia*, and thus would predate at least the diversification of basal eudicots and magnoliids.

Gene lineage evolution, together with expression and functional analyses studies for the *AGL6/AGL13* genes support a major duplication event in the Brassicaceae resulting in the *AGL6* and the *AGL13* clades, as well as distinct roles in flower development in monocots, eudicots, and the Brassicaceae (Viaene et al., 2010). In *Arabidopsis*, both copies are expressed in the ovules, while *AGL6* is restricted to the endothelium, *AGL13* is found in the chalaza, however, *agl6* and *agl13* single null mutants exhibit wild type phenotypes suggesting redundancy (Schauer et al., 2009). In other core-eudicots, like Petunia, *PhAGL6* is expressed in developing petals, carpels and ovules; the *phagl6* mutant does not exhibit noticeable abnormal phenotypes, but it shows a role in petal identity in combination with the two *SEPALLATA* copies *FBP2/5*, as the *fbp2/5 phagl6* triple mutant shows enhanced sepaloid petals (Rijpkema et al., 2009). In grasses, both *osmads6* and *bde* mutants, in rice and maize, respectively, show altered palea identity, abnormal carpels with multiple stigmas and ovules with protruding nucelli (Thompson et al., 2009; Li et al., 2010). These data suggest that *AGL6* grass homologs control palea identity, as well as carpel and ovule development. More recently, studies in orchids have shown that *AGL6* paralogs, together with *AP3* orchid-specific copies have specialized in determining identity of sepal and lateral petals on one side, *versus* identity of the lip on the other. Thus, *AGL6* controls perianth identity and while the PPI AGL61–AGL61–AP31–PI is responsible for the identity of sepals and petals, the combination AGL62–AGL62–AP32–PI controls lip identity (Hsu et al., 2015). The expression pattern here observed for *AfimAGL6*, suggests an early role in establishing floral meristem and sepal identity, together with *AfimFUL* and *AfimSEP* genes, as well as a late role in ovule development. If we consider that the ancestral roles of *AGL6* genes in early diverging angiosperms include both perianth identity ovule development, the lack of mutant phenotypes in ovules in other angiosperms suggest redundancy with other MADS-box ovule -specific transcription factors and the absence of mutant phenotypes in the perianth in the core eudicots suggest redundancy with *SEP* genes for determining floral meristem and perianth identity (Pelaz et al., 2000; Schauer et al., 2009; Rijpkema et al., 2009). The role of *AGL6* genes in controlling perianth identity in early diverging angiosperms and in magnoliids, will have to be explored across a number of species having unipartite sepaloid perianths and bipartite perianths to better assess whether *AGL6* plays a general role in perianth identity, like in *Aristolochia* or a distinct role in sepals *versus* petals or extremely modified petals, like in the case of orchids. As the phylogenetic position of the magnoliids is still uncertain (APG, 2009; Wickett et al., 2014), the perianth-specific identity role here proposed for *AGL6* will have to assessed studying spatio-temporal expression of the *AGL6* orthologs in the ANA grade before extrapolating it to the early radiating angiosperms. In addition, testing protein interactions occurring *in vitro* and *in planta* will be fundamental to identify putative partners of MADS-box floral organ identity proteins with overlapping expression patterns in *Aristolochia*, to propose functional quartets active in floral meristem and perianth identity and to identify protein homo- and heterodimers that are common for eudicots and those that may be unique to the magnoliids.

### Expression Patterns of B-class Homologs in *A. fimbriata* Suggest that they do not Contribute to Perianth Identity

The B-class transcription factors originally included in the model as responsible for petal and stamen identity include the *Arabidopsis APETALA3* and *PISTILLATA* genes (Bowman et al., 1989; Jack et al., 1992) and the *Antirrhinum* orthologs *DEFICIENS* and *GLOBOSA* (Sommer et al., 1990; Tröbner et al., 1992). Both genes in each species have been shown to function as obligate heterodimers to bind DNA and turn on petal specific genes that include a number of transcription factors involved in conical cell differentiation, multicellular trichome formation often accompanied by pigment accumulation, while turning off photosynthetic genes (Goto and Meyerowitz, 1994; Jack et al., 1994; Zachgo et al., 1995; Martin et al., 2002; Mara and Irish, 2008). The heterodimerization and autorregulation of the AP3–PI dimer detected in core eudicot model species have shown to be conserved in monocots, basal eudicots and basal angiosperms, suggesting that the AP3–PI interaction is a central hub in the petal–stamen identity programs in flowering plants (Kanno et al., 2003; Melzer et al., 2014; Hsu et al., 2015). In addition to this interaction, homodimerization has been detected independently for AP3/DEF proteins as well as for PI/GLO proteins; the occurrence of such interactions in gymnosperms has been proposed as a prerequisite to the heterodimerization in angiosperms; however, its biological significance is still poorly understood (Winter et al., 2002a,b; Kanno et al., 2003; Whipple et al., 2004; Melzer et al., 2014).

Although the expression of *AP3* and *PI* as well as the activation of the AP3–PI heterodimer is for the most part restricted to petals and stamens, it occurs also exceptionally in the first floral whorl in a number of monocot species having petaloid sepals, such as in *Agapanthus, Lilium*, and *Tulipa* (van Tunen et al., 1993; Tzeng and Yang, 2001; Kanno et al., 2003; Nakamura et al., 2005). The ectopic expression of the petal/stamen genetic module has been used to explain the petaloid nature of the first whorl in such species in what has been named the sliding borders model, a modified ABC model of flower development (Kanno et al., 2003, 2007). However, as petaloid sepals also occur in the absence of *AP3–PI* coordinated expression in other monocots, like *Asparagus* (Asparagaceae; Park et al., 2003) and *Habenaria* (Orchidaceae; Kim et al., 2007), in basal eudicots like *Aquilegia* (Ranunculaceae; Kramer et al., 2007; Sharma et al., 2011; Sharma and Kramer, 2012), and in a number of core eudicots like *Gerbera* (Asteraceae), *Impatiens* (Balsaminaceae) and *Rhodochiton* (Scrophulariaceae; Geuten et al., 2006; Broholm et al., 2010; Landis et al., 2012), it is likely that petaloid features including pigment and structural color as well as epidermal modifications can be turned on independently of AP3–PI (Landis et al., 2012; Weiss, 2000).

In Aristolochiaceae, *AP3* and *PI* expression are restricted to petals and stamens in *Saruma* and by comparison, in *A. fimbriata*, as well as in *A. manshuriensis* and *A. arborea, AP3* and *PI* only overlap in expression patterns in outer portion of the gynostemium, suggesting that the AP3–PI interaction is critical in stamen identity, but does not play any role in perianth identity (**Figures 9** and **10**; Jaramillo and Kramer, 2004; Horn et al., 2014). This is in accordance with the interpretation of the gynostemium as a fused structure between stamens and the upper portion of the carpels provided by González and Stevenson (2000a). Nevertheless, the genetic bases of the proximal-distal differentiation of the gynoecium-derived tissue forming the different portions of the gynostemium will require full examination of the stamen-carpel identity C-class genes as well as the carpel zonation genes responsible for stigmatic and transmitting tissue that include *SPATULA, HECATE3, CRABS CLAW*, and *NGATHA* (Alvarez and Smyth, 2002; Gremski et al., 2007; Fourquin and Ferrándiz, 2014; Schuster et al., 2015).

Interestingly, in *Aristolochia manshuriensis, A. arborea*, and *A. fimbriata PI* expression correlates with the occurrence of conical cellular differentiation and pigment accumulation in the inner epidermis of the perianth (**Figures 4** and **9**; Jaramillo and Kramer, 2004; Horn et al., 2014). Such epidermal specialization that often includes the formation of multicellular trichomes and osmophores is a unique trait of the diploid species of subgenus *Aristolochia*, to which *A. fimbriata* belongs to, and is lacking from the polyploid species prevalent in subgenus *Siphisia*, to which *A. arborea* and *A. manshuriensis* belong (González and Stevenson, 2000b). These observations generate testable hypothesis about a putative role of PI homodimers that together with other MADS-box perianth expressed genes like *FUL* and *AGL6* are able to effectively activate the genetic circuitry responsible for cellular specialization, pigment accumulation, and nectarial secretion in the inner sepal epidermis.

## Author Contributions

NP-M and FG designed the study, NP-M, HS-B, BAA, and FG acquired, analyzed, and interpreted the data, NP-M, BAA, and FG wrote the manuscript, and all authors revised and approved the final version.

## Conflict of Interest Statement

The authors declare that the research was conducted in the absence of any commercial or financial relationships that could be construed as a potential conflict of interest.

## Footnotes

1 http://www.mbio.ncsu.edu/bioedit/bioedit.html

2 http://mafft.cbrc.jp/alignment/server/
