# Supplementary material for: Flower Development and Perianth Identity Candidate Genes in the Basal Angiosperm Aristolochia fimbriata (Piperales: Aristolochiaceae)
Source: Front Plant Sci. 2015 Dec 11;6:1095. doi: 10.3389/fpls.2015.01095 (PMC4675851; doi:10.3389/fpls.2015.01095)
Supplement: Supplementary file 1 [file Table_1.DOCX]

**Supplementary Table 1.** Primers used for all the experiments

|  | **Primer name** | **Primer sequence (5’ - 3’)** | **Tm (ºC)** |
| --- | --- | --- | --- |
| **RT - PCR** | *AfimFUL* Fwd | CATATGCTGAAGAAATCGAGAACAAG | 58 |
|  | *AfimFUL* Rev | GGATCCGCTGTTCGTTTGCGGGCGGCA |  |
|  | *AfimSEP1* Fwd | GTTGCCTTCAGCTTTCGTGGGAAA | 58 |
|  | *AfimSEP1* Rev | CTTAATACGACTCACTATAGGGGTACCTT |  |
|  | *AfimSEP2*  Fwd | CCGGCAAACCCACACCAAGCTTGG | 58 |
|  | *AfimSEP2*  Rev | CTTAATACGACTCACTATAGGGACCGTA |  |
|  | *AfimAGL6* Fwd | CATATGGGACGAGGACGGGTTGAGCT | 58 |
|  | *AfimAGL6* Rev | GGATCCTCAAAGGACCCATCCCTGAA |  |
|  | *AfimAP3* Fwd | CCATGGGTAGAGGGAAGATCGAGATC | 58 |
|  | *AfimAP3* Rev | GGATCCCTATGCGAGGCGTAGATCAT |  |
|  | *AfimPI* Fwd | GGTGTTCGTGCAAGGCAGATGGAA | 58 |
|  | *AfimPI* Rev | CTTAATACGACTCACTATAGGGCTTTTTC |  |
|  | *AfimAG* Fwd | CGAGGGCCTAAGTTCGATGGGTGCG | 58 |
|  | *AfimAG* Rev | CTTAATACGACTCACTATAGGGGCTGTTT |  |
|  | *AfimSTK* Fwd | TGACTCTCTAAGTTCTTTAACAGTG | 50 |
|  | *AfimSTK* Rev | CTTAATACGACTCACTATAGGGATGAAGT |  |
|  | *AfimACTINA* Fwd | GGTTGGGATGGGTCAGAAGG | 54 |
|  | *AfimACTINA* Rev | ACTTGCCCATCAGGAAGCTC |  |
| **In Situ Hybridization** | *AfimFUL* Fwd (antisense probe) | CTCGATAGCAGGAAGGGCGGTGA | 55 |
|  | *AfimFUL* Rev T7 (antisense probe) | CTTAATACGACTCACTATAGGGCGTCTTCTCCAACAGAGGCAAG |  |
|  | *AfimFUL* Fwd T7 (sense control probe) | CTTAATACGACTCACTATAGGGCTCGATAGCAGGAAGGGCGGTGA | 55 |
|  | *AfimFUL* Rev (sense control probe) | CGTCTTCTCCAACAGAGGCAAG |  |
|  | *AfimAP3* Fwd (antisense probe) | GAAAGTATACGACAGATACCAGG | 58 |
|  | *AfimAP3* Rev (antisense probe) | CTAATACGACTCACTATAGGGAGCATCCTGCAAGTTAGGTTG |  |
|  | *AfimAP3* Fwd T7 (sense control probe) | CTAATACGACTCACTATAGGGGAAAGTATACGACAGATACCA | 55 |
|  | *AfimAP3* Rev (sense control probe) | AGCATCCTGCAAGTTAGGTTG |  |
|  | *AfimAGL6* Fwd (antisense probe) | CCTTCTCCAAGAGGAGGAACGG | 58 |
|  | *AfimAGL6* Rev (antisense probe) | CTAATACGACTCACTATAGGGCGGCTTCAAGCTTGTTTTTC |  |
|  | *AfimAGL6* Fwd T7 (sense control probe) | CTAATACGACTCACTATAGGGAAACCCAGGGATGGTATCA | 55 |
|  | *AfimAGL6* Rev (sense control probe) | TTGACACTCAGTGGTCCAAGATC |  |
|  | *AfimPI* Fwd (antisense probe) | AGTGGAAATTCATGGAAGAGG | 55 |
|  | *AfimPI* Rev (antisense probe) | CTTAATACGACTCACTATAGGGAGTTATAGTAGCAGCTATGATC |  |
